# Supplementary material for: Current state of knowledge on Wolbachia infection among Coleoptera: a systematic review
Source: PeerJ. 2018 Mar 9;6:e4471. doi: 10.7717/peerj.4471 (PMC5846457; doi:10.7717/peerj.4471)
Supplement: Table S1 [file peerj-06-4471-s001.pdf]

Supplementary Table 1. Characterization of *Wolbachia* infection across beetle species and populations based on data in the literature.

Citation	Examined- infected species	taxonomy	Geography	*Wolbachia* genotyping	host genotyping	number of examined populations	number of examined individuals	species level	population level	individuals level	supergroup	effects fon hosts	other effects
Kageyama et al. 2010	*Lasioderma serricorne*	Anobiidae	Japane	wsp	n.n.	n.n.	n.n.	n.n.	n.n.	n.n.	n.n.	n.n.	n.n.
Li et al. 2015	*Lasioderma serricorne*	Anobiidae	Canada,Europe,USA	wsp,MLST	n.n.	5	n.n.	all infected	n.n.	n.n.	n.n.	n.n.	n.n.
Kageyama et al. 2010	*Stegobium paniceum*	Anobiidae	Japane	wsp	n.n.	n.n.	n.n.	n.n.	n.n.	n.n.	n.n.	n.n.	n.n.
Li et al. 2015	*Stegobium paniceum*	Anobiidae	Canada,USA	wsp,MLST	n.n.	n.n.	n.n.	n.n.	n.n.	n.n.	n.n.	n.n.	n.n.
Sontowski et al. 2015	*Agrilus araxenus Iablokoff*	Buprestidae	Armenia	ftsZ	n.n.	n.n.	n.n.	n.n.	n.n.	n.n.	F	n.n.	n.n.
Sontowski et al. 2015	*Agrilus ater*	Buprestidae	France	ftsZ	n.n.	n.n.	n.n.	n.n.	n.n.	n.n.	A	n.n.	n.n.
Sontowski et al. 2015	*Agrilus cuprescens*	Buprestidae	Germany	ftsZ	n.n.	n.n.	n.n.	n.n.	n.n.	n.n.	B	n.n.	n.n.
Sontowski et al. 2015	*Agrilus derasofasciatus*	Buprestidae	Armenia	ftsZ	n.n.	n.n.	n.n.	n.n.	n.n.	n.n.	B	n.n.	n.n.
Sontowski et al. 2015	*Agrilus graminis*	Buprestidae	France	ftsZ	n.n.	n.n.	n.n.	n.n.	n.n.	n.n.	B	n.n.	n.n.
Sontowski et al. 2015	*Agrilus ribesi*	Buprestidae	Germany	ftsZ	n.n.	n.n.	n.n.	n.n.	n.n.	n.n.	B	n.n.	n.n.
Sontowski et al. 2015	*Anthaxia anatolica*	Buprestidae	Armenia	ftsZ	n.n.	n.n.	n.n.	n.n.	n.n.	n.n.	B	n.n.	n.n.
Sontowski et al. 2015	*Capnodis tenebricosa*	Buprestidae	Armenia, France	ftsZ	n.n.	n.n.	n.n.	n.n.	n.n.	n.n.	A	n.n.	n.n.
Sontowski et al. 2015	*Chrysobothris affinis*	Buprestidae	Armenia	ftsZ	n.n.	n.n.	n.n.	n.n.	n.n.	n.n.	A	n.n.	n.n.
Sontowski et al. 2015	*Lamprodila mirifica nadezhdae*	Buprestidae	Armenia	ftsZ	n.n.	n.n.	n.n.	n.n.	n.n.	n.n.	A	n.n.	n.n.
Sontowski et al. 2015	*Sphaerobothris aghababiani*	Buprestidae	Armenia	ftsZ	n.n.	n.n.	n.n.	n.n.	n.n.	n.n.	F	n.n.	n.n.
Sontowski et al. 2015	*Sphenoptera antiqua*	Buprestidae	Armenia	ftsZ	n.n.	n.n.	n.n.	n.n.	n.n.	n.n.	A	n.n.	n.n.
Sontowski et al. 2015	*Trachys minutus*	Buprestidae	Germany	ftsZ	n.n.	n.n.	n.n.	n.n.	n.n.	n.n.	A	n.n.	n.n.
Malloch et al. 2000	*Byturus tomentosus*	Byturidae	Europe	ftsZ	n.n.	34	152	n.n.	24% or 99% infected	97% multiple infected	A	n.n.	n.n.
Heintzman et al. 2014	*Amara alpina*	Carabidae	Arctica	Illumina shot-gun seq	n.n.	n.n.	6	n.n.	n.n.	2 strains	AB	n.n.	n.n.
Kittayapong et al.. 2003	*Chlaenius sp.*	Carabidae	Thailand	ftsZ,wsp	n.n.	1	3	1/3 infected	n.n.	n.n.	B	n.n.	n.n.
Frank et al. 2009	*Pheropsophus aequinoctialis*	Carabidae	S America	n.n.	n.n.	n.n.	n.n.	n.n.	n.n.	n.n.	n.n.	n.n.	n.n.
Aikawa et al. 2009	*Monochamus alternatus*	Cerambycidae	Japan	ftsZ,wsp,16S,groEL	n.n.	2	25	4% population infected	22/25 infected	single ftsZ variant	A	14% of W. genes transferred into autosomes of the host - no living bacteria detected	n.n.
Clark et al. 2001	*Acalymma blandulum*	Chrysomelidae	N America	16S, ftsZ	n.n.	1	20	all infected	n.n.	n.n.	A	n.n.	n.n.
Clark et al. 2001	*Acalymna vittatum*	Chrysomelidae	N America	16S, ftsZ	n.n.	1	20	5% infected	n.n.	n.n.	A	n.n.	n.n.
Werren et al. 1995	*Acromis sparsa*	Chrysomelidae	Panama	ftsZ	28S	n.n.	1	n.n.	n.n.	n.n.	AB	n.n.	n.n.
Pankewitz et al. 2007	*Agelastica alni*	Chrysomelidae	Germany	16S,wsp,ftsZ	n.n.	1	5	all infected	all infected	n.n.	A	n.n.	n.n.
Jäckel et al. 2013	*Altica aenescens*	Chrysomelidae	Germany	ftsZ	COI, ITS2, EF1α, msαts	1	3	n.n.	single strain (3/3 infected)	single infected	A	n.n.	horizontal transmission?
Jäckel et al. 2013	*Altica birmanensis*	Chrysomelidae	Taiwan	ftsZ	COI, ITS2, EF1α, msαts	1	1	n.n.	single strain	single infected	A	n.n.	horizontal transmission?
Jäckel et al. 2013	*Altica brevicollis*	Chrysomelidae	Germany	ftsZ	COI, ITS2, EF1α, msαts	1	1	n.n.	single strain	single infected	A	n.n.	horizontal transmission?
Jäckel et al. 2013	*Altica carinata*	Chrysomelidae	USA	ftsZ	COI, ITS2, EF1α, msαts	1	2	n.n.	single strain	single infected	A	n.n.	horizontal transmission?
Jäckel et al. 2013	*Altica carinthiaca*	Chrysomelidae	Germany	ftsZ	COI, ITS2, EF1α, msαts	1	2	n.n.	single strain	single infected	A	n.n.	horizontal transmission?
Xue et al. 2011	*Altica cirsicola*	Chrysomelidae	East Asia	wsp	COI,ITS2,EF1α	2	23	all infected	all infected	single infected	n.n.	n.n.	n.n.
Jäckel et al. 2013	*Altica engstroemi*	Chrysomelidae	Sweden	ftsZ	COI, ITS2, EF1α, msαts	1	2	n.n.	single strain	single infected	B	n.n.	horizontal transmission?
Jäckel et al. 2013	*Altica ericeti*	Chrysomelidae	Germany	ftsZ	COI, ITS2, EF1α, msαts	1	2	n.n.	single strain	single infected	A	n.n.	horizontal transmission?
Xue et al. 2011	*Altica fragariae*	Chrysomelidae	East Asia	wsp	COI,ITS2,EF1α	4	47	all infected	all infected	single infected	n.n.	n.n.	n.n.
Jäckel et al. 2013	*Altica helianthemi*	Chrysomelidae	Germany	ftsZ	COI, ITS2, EF1α, msαts	1	1	n.n.	single strain	single infected	A	n.n.	horizontal transmission?
Jäckel et al. 2013	*Altica impressicollis*	Chrysomelidae	Germany	ftsZ	COI, ITS2, EF1α, msαts	1	2	n.n.	single strain (5/9 infected)	single infected	A	n.n.	horizontal transmission?
Jäckel et al. 2013	*Altica lythri*	Chrysomelidae	Germany; Poland; France	ftsZ	COI, ITS2, EF1α, msαts	18	50	n.n.	single strain (19/50 infected)	single infected or uninfected females	AB	perfect LD between W. infection and mtDNA haplotype (SS), CI, sex ratio distortion	horizontal transmission?
Jäckel et al. 2013	*Altica oleracea*	Chrysomelidae	Europe	ftsZ	COI, ITS2, EF1α, msαts	4	24	n.n.	single strain (10/24 infected)	single infected or uninfected	A	n.n.	horizontal transmission?
Jäckel et al. 2013	*Altica palustris*	Chrysomelidae	Germany	ftsZ	COI, ITS2, EF1α, msαts	1	9	n.n.	single strain	single infected	A	n.n.	horizontal transmission?
Jäckel et al. 2013	*Altica quercetorum*	Chrysomelidae	Germany	ftsZ	COI, ITS2, EF1α, msαts	1	2	n.n.	two strains	double infected	AB	n.n.	horizontal transmission?
Jäckel et al. 2013	*Altica tamaricis*	Chrysomelidae	France	ftsZ	COI, ITS2, EF1α, msαts	1	2	n.n.	single strain	single infected	n.n.	n.n.	horizontal transmission?
Xue et al. 2011	*Altica viridicyanea*	Chrysomelidae	East Asia	wsp	COI,ITS2,EF1α	3	57	all infected	all infected	single infected	n.n.	n.n.	n.n.
Roehrdanz et al. 2006	*Aphthona nigriscutis*	Chrysomelidae	USA, Canada	wsp,ftsZ	16S	3	214	c. 70% infected	60%-94% infected in populations	single strain	A	SS and reduction of host diversity	n.n.
Takano et al. 2017	*Brontispa longissima*	Chrysomelidae	East Timor	16S (Illumina),MLST	n.n.	2	12	n.n.	n.n.	n.n.	n.n.	CI	identification of new Alphaproteobacteria
Kondo et al. 2011	*Callosobruchus analis*	Chrysomelidae	Britain, Sri Lanka, Bangladesh	wsp,ftsZ	mtDNα	3	8	all infected	n.n.	n.n.	B	n.n.	n.n.
Kageyama et al. 2010	*Callosobruchus analis*	Chrysomelidae	Japane	wsp	n.n.	n.n.	n.n.	n.n.	n.n.	n.n.	n.n.	n.n.	n.n.
Numajiri et al. 2017	*Callosobruchus analis*	Chrysomelidae	Sri Lanca	wsp	COI	n.n.	n.n.	n.n.	n.n.	n.n.	n.n.	CI, fitness decline in infected beetles	n.n.
Kageyama et al. 2010	*Callosobruchus chinensis*	Chrysomelidae	Japane	wsp	n.n.	n.n.	n.n.	n.n.	n.n.	n.n.	n.n.	n.n.	n.n.
Nikoh et al. 2008	*Callosobruchus chinensis*	Chrysomelidae	Japan, Taiwan	wsp,ftsZ	n.n.	8 strains	14	n.n.	n.n.	n.n.	n.n.	30% of W. genes ransferred into autosomes of the host	n.n.
Kondo et al. 2011	*Callosobruchus chinensis*	Chrysomelidae	Asia, Africa	wsp,ftsZ	mtDNα	37	184	all infected	n.n.	n.n.	AB	n.n.	n.n.
Kondo et al. 1999	*Callosobruchus chinensis*	Chrysomelidae	Japan	16S, wsp, ftsZ	mtDNα	6	409	all infected	all infected		n.n.	n.n.	n.n.
Kondo et al. 2002	*Callosobruchus chinensis*	Chrysomelidae	Japan	wsp,ftsZ	n.n.	9	622	all infected	all infected	3 strains, infected: 93.7% triple , 6.1% double, 0.2% single, 1 strain in 90-100% ind.	AB	CI, W. populations (strains) controled by the host	n.n.
Kondo et al. 2005	*Callosobruchus chinensis*	Chrysomelidae	Japan	wsp	n.n.	n.n.	n.n.	n.n.	n.n.	n.n.	AB	n.n.	n.n.
Okayama et al. 2016	*Callosobruchus chinensis*	Chrysomelidae	Japan	n.n.	n.n.	1 (single strain)	n.n.	n.n.	n.n.	n.n.	n.n.	infection affects the life history and sexual selection (CI)	n.n.
Goodacre et al. 2015	*Callosobruchus rhodesianus*	Chrysomelidae	Africa,Asia, Australia,N America	wsp	COI	1	4	n.n.	1/4 infected	n.n.	n.n.	n.n.	n.n.
Kondo et al. 2011	*Callosobruchus latealbus*	Chrysomelidae	China, Taiwan	wsp,ftsZ	mtDNα	3	11	all infected	n.n.	2 strains, 10 ind. single infected, 2 ind. double infected	AB	n.n.	n.n.
Werren et al. 2000	*Charidotella purpurata*	Chrysomelidae	USA	ftsZ,16S	n.n.	n.n.	1	n.n.	n.n.	n.n.	A	n.n.	n.n.
Werren et al. 1995	*Chelymorpha alternans*	Chrysomelidae	Panama	ftsZ	28S	n.n.	3	n.n.	n.n.	n.n.	B	n.n.	n.n.
Keller et al. 2004	*Chelymorpha alternans*	Chrysomelidae	Panama	16S, wsp, ftsZ	COI	24	753	747/753 infected	infected by two strains	99.2% and 53% of beetles infected by 2 strains, single or double infected	n.n.	CI	dry climate decrease infection frequency
Clark et al. 2008	*Chelymorpha alternans*	Chrysomelidae	Panama	16S, wsp	n.n.	2	n.n.	n.n.	n.n.	n.n.	n.n.	W. modification of sperm	n.n.
Werren et al. 1995	*Chersinelina heteropunctata*	Chrysomelidae	Panama	ftsZ	28S	n.n.	1	n.n.	n.n.	n.n.	B	n.n.	n.n.
Kubisz et al. 2012	*Crioceris quaterdecimpunctata*	Chrysomelidae	C,E Europe	ftsZ,hcpA	COI, ITS1	5	25	all infected	all infected	most double infected	AB	n.n.	n.n.
Kolasa et al. 2017	*Crioceris quaterdecimpunctata*	Chrysomelidae	C,E Europe	MLST	COI,ITS1	4	19	all infected	all infected	all double infected	AB	n.n.	host plant transmission (Asparagus)
Kolasa et al. 2017	*Crioceris quinquepunctata*	Chrysomelidae	C,E Europe	MLST	COI,ITS1	2	10	all infected	all infected	all double infected	AB	n.n.	host plant transmission (Asparagus)
Mazur et al. 2014	*Crioceris quinquepunctata*	Chrysomelidae	C,E Europe	ftsZ,hcpA	COI,ITS1	2	10	all infected	all infected	most double infected	AB	n.n.	n.n.
Roehrdanz & Levine 2007	*Diabrotica barberi*	Chrysomelidae	USA	16S, wsp, ftsZ	mtDNα	66	577	all infected	all infected	2 distinct strains	A	CI	n.n.
Roehrdan & Wichmann 2013	*Diabrotica barberi*	Chrysomelidae	USA	wsp	n.n.	66	577	all infected	all infected	5 wsp variants	n.n.	n.n.	n.n.
Clark et al. 2001	*Diabrotica cristata*	Chrysomelidae	N America	16S,ftsZ	n.n.	1	25	12% infected	n.n.	n.n.	A	n.n.	n.n.
Clark et al. 2001	*Diabrotica lemniscata*	Chrysomelidae	N America	16S,ftsZ	n.n.	1	20	all infected	n.n.	n.n.	A	n.n.	n.n.
Clark et al. 2001	*Diabrotica virgifera virgifera*	Chrysomelidae	N America	16S,ftsZ	n.n.	1	40	all infected	n.n.	n.n.	A	possible CI	n.n.
Giordano et al. 1997	*Diabrotica virgifera virgifera*	Chrysomelidae	N America	16S,ftsZ	n.n.	15	60	13/15 populations infected	n.n.	n.n.	A	CI	n.n.
Barr et al. 2010	*Diabrotica virgifera virgifera*	Chrysomelidae	N America	n.n.	n.n.	n.n.	n.n.	n.n.	n.n.	n.n.	n.n.	n.n.	W. down-regulate defence genes in maize
Kittayapong et al.. 2003	*Dicladispa armigera*	Chrysomelidae	Thailand	ftsZ,wsp	n.n.	1	12	1/12 infected	n.n.	n.n.	B	n.n.	n.n.
Pankewitz et al. 2007	*Galeruca tanaceti*	Chrysomelidae	Germany	16S,wsp,ftsZ	n.n.	1	5	all infected	all infected	n.n.	A	n.n.	n.n.
Jäckel et al. 2013	*Hermaeophaga mercurialis*	Chrysomelidae	n.n.	ftsZ	COI,ITS2,EF1α	1	1	n.n.	n.n.	n.n.	A	n.n.	n.n.
Kondo et al. 2011	*Megabruchidius sophorae*	Chrysomelidae	Japan	wsp,ftsZ	mtDNA	1	8	1/8 infected	n.n.	n.n.	B	n.n.	n.n.
Sintupachee et al. 2006	*Monolepta signata*	Chrysomelidae	Thailand	ftsZ,wsp	n.n.	1	19	1/19 infected	n.n.	n.n.	n.n.	n.n.	n.n.
Chafee et al. 2009	*Neochlamisus bebbianae*	Chrysomelidae	N America	MLST	n.n.	n.n.	n.n.	n.n.	n.n.	n.n.	B	n.n.	n.n.
Montagna et al. 2014	*Oreina bidentata*	Chrysomelidae	Italy	16S,wsp,MLST	COI	1	1	all infected	all infected	single strain	A	n.n.	n.n.
Montagna et al. 2014	*Oreina cacaliae*	Chrysomelidae	Italy	16S,wsp,MLST	COI	1	15	all infected	all infected	single strain	A	n.n.	n.n.
Montagna et al. 2014	*Oreina elongate*	Chrysomelidae	Italy	16S,wsp,MLST	COI	2	3	all infected	all infected	single strain	A	n.n.	n.n.
Montagna et al. 2014	*Oreina liturata*	Chrysomelidae	Italy	16S,wsp,MLST	COI	1	1	all infected	all infected	single strain	A	n.n.	n.n.
Montagna et al. 2014	*Oreina speciosa*	Chrysomelidae	Italy	16S,wsp,MLST	COI	3	5	all infected	all infected	single strain	A	n.n.	n.n.
Sintupachee et al. 2006	*Phyllotreta sp.*	Chrysomelidae	Thailand	ftsZ,wsp	n.n.	1	4	1/4 infected	n.n.	n.n.	n.n.	n.n.	n.n.
Jäckel et al. 2013	*Podagrica fuscipes*	Chrysomelidae	n.n.	ftsZ	COI,ITS2,EF1α	1	1	n.n.	n.n.	n.n.	B	n.n.	n.n.
Yun et al. 2011	*Propylea japonica*	Chrysomelidae	China	wsp	n.n.	n.n.	44	n.n.	n.n.	n.n.	B	n.n.	n.n.
Werren et al. 1995	*Priocera sp.*	Cleridae	Panama	ftsZ	28S	n.n.	1	n.n.	n.n.	n.n.	AB	n.n.	n.n.
Majerus et al. 2000	*Adalia bipunctata*	Coccinellidae	Russia	wsp	n.n.	1	110 (40 lines)	2 lines infected	n.n.	2 strains	B	killing of male embryo	coexistence with Rickettsia & Spiroplasma
Hurst et al. 1999	*Adalia bipunctata*	Coccinellidae	Russia	ftsZ,wsp,16S	n.n.	1	5	n.n.	all infected	n.n.	B	n.n.	n.n.
Elnagdy et al. 2013	*Adalia bipunctata*	Coccinellidae	Russia	n.n.	n.n.	3 lines	40	n.n.	n.n.	n.n.	n.n.	n.n.	n.n.
Weinert et al. 2007	*Calvia quattuordecimguttata*	Coccinellidae	Germany	wsp	n.n.	n.n.	>20	n.n.	4% of males infected	n.n.	n.n.	n.n.	coexistence with Rickettsia
Weinert et al. 2007	*Chilocorus bipustulatus*	Coccinellidae	Italy	wsp	n.n.	n.n.	>20	n.n.	8% of females infected	n.n.	n.n.	n.n.	coexistence with Spiroplasma
Weinert et al. 2007	*Coccidula rufa*	Coccinellidae	Germany	wsp	n.n.	n.n.	>20	n.n.	78% of females & 8% of males infected	n.n.	n.n.	n.n.	coexistence with Rickettsia
Weinert et al. 2007	*Coccinella septempunctata*	Coccinellidae	Britain	wsp	n.n.	n.n.	>20	n.n.	5% of females infected	n.n.	n.n.	n.n.	coexistence with Rickettsia
Pourali et al.. 2009	*Coccinella trifasciata*	Coccinellidae	Iran	16S,wsp	n.n.	1	6	2/6 infected	n.n.	n.n.	A	n.n.	n.n.
Elnagdy et al. 2013	*Coccinella undecimpunctata*	Coccinellidae	Egypt	n.n.	n.n.	6 lines	40	n.n.	n.n.	n.n.	n.n.	n.n.	n.n.
Jeyaprakash & Hoy 2000	*Coleomegilla maculata*	Coccinellidae	USA	ftsZ	n.n.	n.n.	n.n.	n.n.	n.n.	n.n.	B	n.n.	n.n.
Nirgianaki et al.. 2003	*Delphastus pusillus*	Coccinellidae	USA	16S,wsp	n.n.	1	9	all infected	n.n.	n.n.	n.n.	n.n.	n.n.
Weinert et al. 2007	*Halyzia sedecimguttata*	Coccinellidae	Germany	wsp	n.n.	n.n.	>20	n.n.	2% of females infected	n.n.	n.n.	n.n.	coexistence with Rickettsia
Dudek et al. 2017	*Harmonia axyridis*	Coccinellidae	Poland	16S NGS (Illumina)	n.n.	1	15	n.n.	1/15 specimens infected	n.n.	n.n.	n.n.	coexistence with Rickettsia & Spiroplasma
Goryachevaa et al. 2015	*Harmonia axyridis*	Coccinellidae	Europe, Japan, USA	MSLT, wsp	n.n.	13	375	4 out of 13 infected	n.n.	n.n.	AB	n.n.	n.n.
Weinert et al. 2007	*Rhizobius litura*	Coccinellidae	Germany	wsp	n.n.	n.n.	>20	n.n.	89% of females & 5% of males infected	n.n.	n.n.	n.n.	coexistence with Rickettsia
Rodriguer et al. 2010a	*Aramigus conirostris*	Curculionidae	S America	MLST	COI	n.n.	1	all infected	all infected	single strain	B	only parthenogenetic or presumed parthenogenetic species infected	n.n.
Rodriguer et al. 2010a	*Aramigus tessellatus*	Curculionidae	S America	MLST	COI	n.n.	2	all infected	all infected	single strain	B	n.n.	n.n.
Toju et al. 2013	*Archarius roelofsi*	Curculionidae	Asia	16S	n.n.	3	3	n.n.	n.n.	n.n.	n.n.	n.n.	n.n.
Rodriguer et al. 2010a	*Atrichonotus taeniatulus*	Curculionidae	S America	MLST	COI	n.n.	1	all infected	all infected	single strain	B	n.n.	n.n.
Campbell et al. 1992	*Bangasternus orientalis*	Curculionidae	Italy	16S	n.n.	n.n.	n.n.	n.n.	n.n.	n.n.	n.n.	n.n.	n.n.
Lachowska-Cierlik et al. 2010	*Barypeithes pellucidus*	Curculionidae	Poland	wsp,16S,ftsZ,hcpA	COI	1	1	n.n.	n.n.	n.n.	A	n.n.	n.n.
Lachowska-Cierlik et al. 2010	*Brachyderes incanus*	Curculionidae	Poland	wsp,16S,ftsZ,hcpA	COI	1	1	n.n.	n.n.	n.n.	AB	n.n.	n.n.
Piper et al. 2001	*Cathormiocerus britannicus*	Curculionidae	England	16S	ITS2	3	5	all infected	all infected	n.n.	n.n.	n.n.	n.n.
Piper et al. 2001	*Cathormiocerus myrmecophilus*	Curculionidae	England	16S	ITS2	3	3	all infected	all infected	n.n.	n.n.	n.n.	n.n.
Floate et al. 2011	*Ceutorhynchus obstrictus*	Curculionidae	Europe, N America	MSLT,wsp	COI	11	64	all infected	all infected	n.n.	n.n.	n.n.	n.n.

Zhang et al. 2010	*Conotrachelus nenuphar*	Curculionidae	USA	wsp,ftsZ	COI	12	94	98% infected	n.n.	3 strains (double and single infected)	AB	possible CI	n.n.
Werren et al. 1995	*Cossomus sp.*	Curculionidae	Panama	ftsZ	28S	n.n.	1	n.n.	n.n.	n.n.	AB	n.n.	n.n.
Zchori-Fein et al. 2006	*Coccotrypes dactyliperda*	Curculionidae	Israel	16S	n.n.	1	20	n.n.	all infected	n.n.	n.n.	role in oogenesis	n.n.
Toju & Fukatsu 2011	*Curculio sikkimensis*	Curculionidae	Japane	16S	EF1α	55	968	n.n.	82.5% infected	n.n.	n.n.	n.n.	coexistence with Rickettsia & Spiroplasma
Toju et al. 2013	*Curculio hachijoensis*	Curculionidae	Asia	16S	n.n.	1	2	n.n.	n.n.	n.n.	n.n.	n.n.	n.n.
Toju et al. 2013	*Curculio hilgendorfi*	Curculionidae	Asia	16S	n.n.	2	3	n.n.	n.n.	n.n.	n.n.	n.n.	n.n.
Toju et al. 2013	*Curculio morimotoi*	Curculionidae	Asia	16S	n.n.	1	2	n.n.	n.n.	n.n.	n.n.	n.n.	n.n.
Toju et al. 2013	*Curculio okumai*	Curculionidae	Asia	16S	n.n.	2	4	n.n.	n.n.	n.n.	n.n.	n.n.	n.n.
Kajtoch et al. 2017	*Cyanapion afer*	Curculionidae	Poland	MLST	COI, ITS2, EF1α	5	10	all infected	all infected	2 strains	A	n.n.	n.n.
Kajtoch et al. 2017	*Cyanapion gyllenhalii*	Curculionidae	Poland	MLST	COI, ITS2, EF1α	6	12	all infected	all infected	single strain	A	n.n.	n.n.
Kajtoch et al. 2017	*Cyanapion spencii*	Curculionidae	Poland	MLST	COI, ITS2, EF1α	6	12	n.n.	5/6 infected	2 strains	AB	n.n.	n.n.
Werren et al. 2000	*Cyrtepistomus castaneus*	Curculionidae	USA	ftsZ,16S	n.n.	n.n.	1	n.n.	n.n.	n.n.	A	n.n.	n.n.
Lachowska-Cierlik et al. 2010	*Dorytomus carpathicus*	Curculionidae	Poland	wsp,16S,ftsZ,hcpA	COI	1	1	n.n.	n.n.	n.n.	A	n.n.	n.n.
Lachowska-Cierlik et al. 2010	*Dorytomus rufatus*	Curculionidae	Poland	wsp,16S,ftsZ,hcpA	COI	1	1	n.n.	n.n.	n.n.	AB	n.n.	n.n.
Lachowska-Cierlik et al. 2010	*Ellescus bipunctatus*	Curculionidae	Poland	wsp,16S,ftsZ,hcpA	COI	1	1	n.n.	n.n.	n.n.	B	n.n.	n.n.
Rodriguer et al. 2010a	*Eurymetopus fallax*	Curculionidae	S America	MLST	COI	n.n.	1	all infected	all infected	single strain	B	n.n.	n.n.
Rodriguer et al. 2010a	*Eurymetopus globosus*	Curculionidae	S America	MLST	COI	n.n.	1	all infected	all infected	single strain	B	n.n.	n.n.
Lachowska-Cierlik et al. 2010	*Eusomus ovulum*	Curculionidae	Poland	wsp,16S,ftsZ,hcpA	COI	1	1	n.n.	-	n.n.	A	n.n.	n.n.
Mazur et al. 2016	*Eusomus ovulum*	Curculionidae	C,SE,E Europe	MLST	COI, ITS2, EF1α	28	65	all infected	all infected, single strain	all infected, single strain	A	SS, possible pathernogenesis induction	n.n.
Kawasaki et al. 2016	*Euwallacea interjectus*	Curculionidae	Japan	wsp,MLST	COI, EF1α	n.n.	20	n.n.	100% infected	single infected	A	haplodiploidy induction uncertain	horizontal transmission?
Kawasaki et al. 2016	*Euwallacea validus*	Curculionidae	Japan	wsp,MLST	COI, EF1α	n.n.	21	n.n.	75% infected	single infected	A	haplodiploidy induction uncertain	horizontal transmission?
Kittayapong et al.. 2003	*Hydronomidius molitor*	Curculionidae	Thailand	ftsZ,wsp	n.n.	1	1	n.n.	n.n.	n.n.	A	n.n.	n.n.
Berasategui et al. 2016	*Hylobius abietis*	Curculionidae	Europe	16S NGS(454)	n.n.	6	54	all infected	0.2-100% abundance, 28-73% of reads	n.n.	n.n.	n.n.	n.n.
O'Neill et al. 1992	*Hypera postica*	Curculionidae	n.n.	16S	n.n.	n.n.	n.n.	n.n.	n.n.	n.n.	n.n.	n.n.	n.n.
Iwase et al.. 2015	*Hypera postica*	Curculionidae	Japane, Europe	wsp,ftsZ,coxA,hpcA	CytB	11	242	5/11 populations infected	0-100% infected	2 strains	B	n.n.	n.n.
Mariño et al. 2017	*Hypothenemus hampei*	Curculionidae	Puerto Rico	wsp	n.n.	1	n.n.	n.n.	n.n.	n.n.	n.n.	CI	W. as biocontrol agent
Vega et al. 2002	*Hypothenemus hampei*	Curculionidae	S & C America, Asia, Africa	wsp	n.n.	17 countries	n.n.	n.n.	11/17 populations infected	possibly only single strain	B	possible sex determination	n.n.
Toju et al. 2013	*Koreoculio minutissimus*	Curculionidae	Asia	16S	n.n.	2	3	n.n.	n.n.	n.n.	n.n.	n.n.	n.n.
Chen et al. 2012	*Lissorhoptrus oryzophilus*	Curculionidae	China, Texas	wsp,MLST	n.n.	6	127	all infected	all infected	all infected	B	W. necessary for oocyte production	n.n.
Lu et al. 2014	*Lissorhoptrus oryzophilus*	Curculionidae	China, Texas	16S	n.n.	5	1421	all infected	all infected	99 infected	n.n.	n.n.	n.n.
Rodriguer et al. 2010a	*Mimographus ocellatus*	Curculionidae	S America	MLST	COI	n.n.	1	all infected	all infected	single infected	B	n.n.	n.n.
Prakash & Puttaraju 2006	*Myllocerus discolor*	Curculionidae	India	wsp	n.n.	n.n.	n.n.	n.n.	n.n.	n.n.	n.n.	n.n.	n.n.
Rodriguer et al. 2010a	*Naupactus ambiguus*	Curculionidae	S America	MLST	COI	n.n.	3	all infected	all infected	single infected	B	n.n.	n.n.
Rodriguer et al. 2010a	*Naupactus cervinus*	Curculionidae	S America	MLST	COI	n.n.	20	all infected	all infected	single infected	B	n.n.	n.n.
Rodriguero et al. 2010b	*Naupactus cervinus*	Curculionidae	S America	16S, wsp, coxA, fbpA	COI, ITS1n.n.18S	39	309	all infected	all infected	single infected	B	LD	host bottleneck caused by W.
Rodriguer et al. 2010a	*Naupactus condecoratus*	Curculionidae	S America	MLST	COI	n.n.	1	all infected	all infected	single infected	B	n.n.	n.n.
Rodriguer et al. 2010a	*Naupactus cyphoides*	Curculionidae	S America	MLST	COI	n.n.	1	all infected	all infected	single infected	B	n.n.	n.n.
Rodriguer et al. 2010a	*Naupactus dissimilis*	Curculionidae	S America	MLST	COI	n.n.	1	all infected	all infected	single infected	B	n.n.	n.n.
Rodriguer et al. 2010a	*Naupactus leucoloma*	Curculionidae	S America	MLST	COI	n.n.	3	all infected	all infected	single infected	B	n.n.	n.n.
Rodriguer et al. 2010a	*Naupactus minor*	Curculionidae	S America	MLST	COI	n.n.	3	all infected	all infected	single infected	B	n.n.	n.n.
Rodriguer et al. 2010a	*Naupactus peregrinus*	Curculionidae	S America	MLST	COI	n.n.	1	all infected	all infected	single infected	B	n.n.	n.n.
Rodriguer et al. 2010a	*Naupactus purpureoviolaceus*	Curculionidae	S America	MLST	COI	n.n.	1	all infected	all infected	single infected	B	n.n.	n.n.
Rodriguer et al. 2010a	*Naupactus tremolerasi*	Curculionidae	S America	MLST	COI	n.n.	1	all infected	all infected	single infected	B	n.n.	n.n.
Rodriguer et al. 2010a	*Naupactus verecundus*	Curculionidae	S America	MLST	COI	n.n.	1	all infected	all infected	single infected	B	n.n.	n.n.
Lachowska-Cierlik et al. 2010	*Otiorhynchus coecus*	Curculionidae	Poland	wsp,16S,ftsZ,hcpA	COI	1	1	n.n.	n.n.	n.n.	AB	n.n.	n.n.
Stenberg & Lundmark 2004	*Otiorhynchus scaber*	Curculionidae	Austria, Slovenia	16S	COI,COII,CytB	2	28	all infected	infected: 5/10 ♂ , 2/7 sexual ♀, 0 of clonal ♀	n.n.	B	n.n.	n.n.
Lachowska-Cierlik et al. 2010	*Otiorhynchus singularis*	Curculionidae	Poland	wsp,16S,ftsZ,hcpA	COI	1	1	n.n.	n.n.	n.n.	AB	n.n.	n.n.
Son et al. 2008	*Otiorhynchus sulcatus*	Curculionidae	USA	wsp	n.n.	5	96	all infected	all infected	n.n.	B	W. necessary for egg developnment	n.n.
Rodriguer et al. 2010a	*Pantomorus auripes*	Curculionidae	S America	MLST	COI	n.n.	3	all infected	all infected	single infected	B	n.n.	n.n.
Rodriguer et al. 2010a	*Pantomorus cinerosus*	Curculionidae	S America	MLST	COI	n.n.	2	all infected	all infected	single infected	B	n.n.	n.n.
Rodriguer et al. 2010a	*Pantomorus viridisquamosus*	Curculionidae	S America	MLST	COI	n.n.	2	all infected	all infected	single infected	B	n.n.	n.n.
Lachowska-Cierlik et al. 2010	*Paophilus aflatus*	Curculionidae	Poland	wsp,16S,ftsZ,hcpA	COI	1	1	n.n.	n.n.	n.n.	A	n.n.	n.n.
Lachowska-Cierlik et al. 2010	*Parafoucartia squamulata*	Curculionidae	Poland	wsp,16S,ftsZ,hcpA	COI	1	1	n.n.	n.n.	n.n.	A	n.n.	n.n.
Arthofer et al. 2009	*Pityogenes chalcographus*	Curculionidae	C,E,N,S Europe	wsp, MLST	COI, EF1α	31	344	35.5% infected	0-100% infected	3 strains (91% - A, 9% - B)(95% single infected, 5% double infected)	AB	n.n.	bacteria loss and horizontal transfer
Lachowska-Cierlik et al. 2010	*Polydrusus inustus*	Curculionidae	Poland	wsp,16S,ftsZ,hcpA	COI	1	1	n.n.	n.n.	n.n.	A	n.n.	n.n.
Kajtoch et al. 2012	*Polydrusus inustus*	Curculionidae	C,E Europe	wsp,16S,ftsZ,hcpA	COI, ITS2,EF1α	9	40	all infected	all infected, 2 closely related strains	all infected, 2 closely related strains	A	probably SS	n.n.
Lachowska-Cierlik et al. 2010	*Polydrusus mollis*	Curculionidae	Poland	wsp,16S,ftsZ,hcpA	COI	1	1	n.n.	n.n.	n.n.	AB	n.n.	n.n.
Kajtoch et al. 2012	*Polydrusus pilifer*	Curculionidae	Caucasus	wsp,16S,ftsZ,hcpA	COI, ITS2,EF1α	1	5	all infected	all infected	single infected	A	probably SS	n.n.
Lachowska-Cierlik et al. 2010	*Polydrusus pilosus*	Curculionidae	Poland	wsp,16S,ftsZ,hcpA	COI	1	1	n.n.	n.n.	n.n.	AB	n.n.	n.n.
Campbell et al. 1992	*Rhinocyllus conicus*	Curculionidae	Italy	16S	n.n.	n.n.	n.n.	n.n.	n.n.	n.n.	F	n.n.	n.n.
Toševski et al. 2015	*Rhinusa pilosa*	Curculionidae	Europe	coxA	COI, 16S,EF1α	6	47	n.n.	80.8% ind. Infected	n.n.	n.n.	n.n.	n.n.
Toševski et al. 2015	*Rhinusa rara*	Curculionidae	Europe	coxA	COI, 16S,EF1α	5	10	n.n.	60% ind. Infected	n.n.	n.n.	n.n.	n.n.
Lachowska-Cierlik et al. 2010	*Sciaphobus rubi*	Curculionidae	Poland	wsp,16S,ftsZ,hcpA	COI	1	1	n.n.	n.n.	n.n.	B	n.n.	n.n.
White et al. 2015	*Sitona obsoletus*	Curculionidae	New Zealand	16S(454),MLST	n.n.	2	36	all infected	all infected	W. = 31-72% of bacteria reads	A	n.n.	coexistence with Rickettsia
Heddi et al. 1999	*Sitophilus granarius*	Curculionidae	France	16S	n.n.	3	30	2 out of 3 infected	40% infected	n.n.	n.n.	n.n.	n.n.
Heddi et al. 1999	*Sitophilus oryzae*	Curculionidae	Africa,Asia, Australia,N America	16S	n.n.	10	100	7 out of 10 infected	61% infected	n.n.	B	n.n.	n.n.
Carvalho et al. 2014	*Sitophilus oryzae*	Curculionidae	America S, N, Asia, Australia	16S	16S	16	n.n.	n.n.	7/16 infected	n.n.	n.n.	n.n.	horizontal transmission
Li et al. 2015	*Sitophilus oryzae*	Curculionidae	Canada	wsp,MLST	n.n.	7	n.n.	1/6 populations infected	n.n.	n.n.	n.n.	n.n.	n.n.
Werren et al. 1995	*Sitophilus oryzae*	Curculionidae	Panama	ftsZ	28S	n.n.	1	n.n.	n.n.	n.n.	B	n.n.	n.n.
Carvalho et al. 2014	*Sitophilus zaemais*	Curculionidae	America S, N, Asia, Australia	16S	16S	11	n.n.	n.n.	9/11 infected	n.n.	n.n.	n.n.	horizontal transmission
Kageyama et al. 2010	*Sitophilus zaemais*	Curculionidae	Japane	wsp	n.n.	n.n.	n.n.	n.n.	n.n.	n.n.	n.n.	n.n.	n.n.
Li et al. 2015	*Sitophilus zaemais*	Curculionidae	Thailand	wsp,MLST	n.n.	3	n.n.	2/3 populations infected	n.n.	n.n.	n.n.	n.n.	n.n.
Heddi et al. 1999	*Sitophilus zaemais*	Curculionidae	Asia, Africa, America,	16S	n.n.	10	100	4 out of 10 infected	40% infected	n.n.	n.n.	n.n.	n.n.
White et al. 2015	*Steriphus variabilis*	Curculionidae	New Zealand	16S(454),MLST	n.n.	1	18	all infected	all infected	W. = 8-12% of bacteria reads	B	n.n.	n.n.
Lachowska-Cierlik et al. 2010	*Strophosoma capitatum*	Curculionidae	Poland	wsp,16S,ftsZ,hcpA	COI	1	1	n.n.	n.n.	n.n.	B	n.n.	n.n.
Lachowska-Cierlik et al. 2010	*Strophosoma melanogrammum*	Curculionidae	Poland	wsp,16S,ftsZ,hcpA	COI	1	1	n.n.	n.n.	n.n.	B	n.n.	n.n.
Kawasaki et al. 2016	*Taphrorychus bicolor*	Curculionidae	Austria	wsp,MLST	COI,EF1α	n.n.	5	n.n.	80% infected	single infected	B	n.n.	horizontal transmission?
Kawasaki et al. 2016	*Xyleborinus schaufussi*	Curculionidae	Japan	wsp,MLST	COI,EF1α	n.n.	11	n.n.	85% infected	single infected	A	haplodiploidy induction uncertain	horizontal transmission?
Kawasaki et al. 2016	*Xyleborus dispar*	Curculionidae	Austria	wsp,MLST	COI,EF1α	n.n.	5	n.n.	100% infected	6 strains, ind. multiple infected	A	haplodiploidy induction uncertain	horizontal transmission?
Kawasaki et al. 2016	*Xyleborus seiryorensis*	Curculionidae	Japan	wsp,MLST	COI,EF1α	n.n.	7	n.n.	100% infected	single infected	A	haplodiploidy induction uncertain	horizontal transmission?
Kawasaki et al. 2016	*Xylosandrus crassiusculus*	Curculionidae	Japan	wsp,MLST	COI,EF1α	n.n.	47	n.n.	95% infected	single infected	A	haplodiploidy induction uncertain	horizontal transmission?
Kawasaki et al. 2016	*Xylosandrus germanus*	Curculionidae	Japane	wsp,MLST	COI	9	120	all infected	all infected, 5 strains, 1-4 strains/population	9 allele combinations	A	multi-directional CI	n.n.
Kawasaki et al. 2016	*Xylosandrus germanus*	Curculionidae	Germany, Hungary	wsp,MLST	COI,EF1α	n.n.	130	n.n.	100% infected	4 strains, ind. multiple infected	A	haplodiploidy induction uncertain	horizontal transmission?
Kageyama et al. 2010	*Anthrenus verbasci*	Dermestidae	Japane	wsp	n.n.	n.n.	n.n.	n.n.	n.n.	n.n.	n.n.	n.n.	n.n.
Li et al. 2015	*Attagenus unicolor*	Dermestidae	USA	wsp,MLST	n.n.	n.n.	n.n.	n.n.	n.n.	n.n.	n.n.	n.n.	
Li et al. 2015	*Dermestes lardarius*	Dermestidae	Canada	wsp,MLST	n.n.	n.n.	n.n.	n.n.	n.n.	n.n.	n.n.	n.n.	
García-Vázquez & Ribera 2016	*Deronectes angelinii*	Dytiscidae	Italy	wsp	COI,16S,NADH,H3,Wg	1	1	n.n.	n.n.	n.n.	B	n.n.	n.n.
García-Vázquez & Ribera 2016	*Deronectes aubei*	Dytiscidae	Italy, Germany, Spain, France	wsp	COI,16S,NADH,H3,Wg	5	15	8/15 infected	n.n.	n.n.	A	n.n.	n.n.
García-Vázquez & Ribera 2016	*Deronectes delarouzei*	Dytiscidae	Spain	wsp	COI,16S,NADH,H3,Wg	4	4	all infected	n.n.	n.n.	A	n.n.	n.n.
García-Vázquez & Ribera 2016	*Deronectes moestus*	Dytiscidae	Greece, Italy, Spain, Marocco	wsp	COI,16S,NADH,H3,Wg	21	21	11/21 infected	n.n.	n.n.	A,B	n.n.	n.n.
García-Vázquez & Ribera 2016	*Deronectes semirufus*	Dytiscidae	Italy	wsp	COI,16S,NADH,H3,Wg	5	6	all infected	n.n.	n.n.	A,B	n.n.	n.n.
Sontowski et al. 2015	*Agabus bipustulatus*	Dytiscidae	Germany	ftsZ	n.n.	n.n.	n.n.	n.n.	n.n.	n.n.	A	n.n.	n.n.
Sontowski et al. 2015	*Copelatus haemorrhoidalis*	Dytiscidae	Germany	ftsZ	n.n.	n.n.	n.n.	n.n.	n.n.	n.n.	A	n.n.	n.n.
Sontowski et al. 2015	*Hygrotus versicolor*	Dytiscidae	Germany	ftsZ	n.n.	n.n.	n.n.	n.n.	n.n.	n.n.	B	n.n.	n.n.
Sontowski et al. 2015	*Gyrinus suffriani*	Gyrinidae	Germany	ftsZ	n.n.	n.n.	n.n.	n.n.	n.n.	n.n.	A	n.n.	n.n.
Sontowski et al. 2015	*Haliplus fluviatilis*	Haliplidae	Germany	ftsZ	n.n.	n.n.	n.n.	n.n.	n.n.	n.n.	A	n.n.	n.n.
Sontowski et al. 2015	*Hydraena brachymera*	Hydraenidae	n.n.	MLST	18S,28S,COI	n.n.	n.n.	n.n.	n.n.	n.n.	A	n.n.	n.n.
Sontowski et al. 2015	*Hydraena corinna*	Hydraenidae	n.n.	MLST	18S,28S,COI	n.n.	n.n.	n.n.	n.n.	n.n.	A	n.n.	n.n.
Sontowski et al. 2015	*Hydraena gracilis*	Hydraenidae	n.n.	MLST	18S,28S,COI	n.n.	n.n.	n.n.	n.n.	n.n.	A	n.n.	n.n.
Sontowski et al. 2015	*Hydraena iberica*	Hydraenidae	n.n.	MLST	18S,28S,COI	n.n.	n.n.	n.n.	n.n.	n.n.	A	n.n.	n.n.
Sontowski et al. 2015	*Hydraena inapicipalpis*	Hydraenidae	n.n.	MLST	18S,28S,COI	n.n.	n.n.	n.n.	n.n.	n.n.	A	n.n.	n.n.
Sontowski et al. 2015	*Hydraena morio*	Hydraenidae	n.n.	MLST	18S,28S,COI	n.n.	n.n.	n.n.	n.n.	n.n.	A	n.n.	n.n.
Sontowski et al. 2015	*Hydraena pygmaea*	Hydraenidae	n.n.	MLST	18S,28S,COI	n.n.	n.n.	n.n.	n.n.	n.n.	A	n.n.	n.n.
Sontowski et al. 2015	*Hydraena riberai*	Hydraenidae	n.n.	MLST	18S,28S,COI	n.n.	n.n.	n.n.	n.n.	n.n.	A	n.n.	n.n.
Sontowski et al. 2015	*Limnebius atomus*	Hydraenidae	n.n.	MLST	18S,28S,COI	n.n.	n.n.	n.n.	n.n.	n.n.	B	n.n.	n.n.
Sontowski et al. 2015	*Limnebius truncatellus*	Hydraenidae	n.n.	MLST	18S,28S,COI	n.n.	n.n.	n.n.	n.n.	n.n.	A	n.n.	n.n.
Sontowski et al. 2015	*Ochthebius exculptus*	Hydraenidae	n.n.	MLST	18S,28S,COI	n.n.	n.n.	n.n.	n.n.	n.n.	B	n.n.	n.n.
Sontowski et al. 2015	*Ochthebius meridionales*	Hydraenidae	n.n.	MLST	18S,28S,COI	n.n.	n.n.	n.n.	n.n.	n.n.	B	n.n.	n.n.
Sontowski et al. 2015	*Ochthebius minimus*	Hydraenidae	n.n.	MLST	18S,28S,COI	n.n.	n.n.	n.n.	n.n.	n.n.	B	n.n.	n.n.
Sontowski et al. 2015	*Ochthebius tivelunus*	Hydraenidae	n.n.	MLST	18S,28S,COI	n.n.	n.n.	n.n.	n.n.	n.n.	A	n.n.	n.n.
Sontowski et al. 2015	*Berosus luridus*	Hydrophilidae	n.n.	ftsZ	n.n.	n.n.	n.n.	n.n.	n.n.	n.n.	A	n.n.	n.n.
Sontowski et al. 2015	*Hydrochus sp.*	Hydrophilidae	n.n.	ftsZ	n.n.	n.n.	n.n.	n.n.	n.n.	n.n.	A	n.n.	n.n.
Jeong et al. 2009	*Luciola unmunsana*	Lampyridae	Korea	16S,wsp	n.n.	5	10	n.n.	80% infected	n.n.	A	n.n.	n.n.
Werren et al. 2000	*Epicauta cinerea*	Meloidae	USA	ftsZ,16S	n.n.	n.n.	1	n.n.	n.n.	n.n.	B	n.n.	n.n.
Perotti et al. 2016	*Micromalthus debilis*	Micromalthidae	USA	16S,wsp	n.n.	1	62	n.n.	3/62 infected	n.n.	n.n.	parthenogenetic paedogenesis induction?	coexistence with Rickettsia
Sontowski et al. 2015	*Noterus clavicornis*	Noteridae	Germany	ftsZ	n.n.	n.n.	n.n.	n.n.	n.n.	n.n.	A	n.n.	n.n.
Jensen 2011	*Popillia japonica*	Scarabaeidae	Japane	wsp	COI	5 plant hosts	182	n.n.	11.7% infected	n.n.	n.n.	possible CI	n.n.

Sintupachee et al. 2006	*Onthophagus vaulongeri*	Scarabaeidae	Thailand	ftsZ,wsp	n.n.	1	6	1/6 infected	n.n.	n.n.	n.n.	n.n.	n.n.
Yun et al. 2011	*Paederus fuscipes*	Staphylinidae	China	wsp	n.n.	n.n.	7	n.n.	n.n.	n.n.	B	n.n.	n.n.
Bili et al. 2016	*Aleochara bilineata*	Staphylinidae	France	16S NGS(454), fbpA	n.n.	2	40	n.n.	n.n.	n.n.	A	n.n.	coexistence with Rickettsia & Spiroplasma
Bili et al. 2016	*Aleochara bipustulata*	Staphylinidae	France	16S NGS(454), fbpA	n.n.	2	40	n.n.	n.n.	n.n.	B	n.n.	coexistence with Spiroplasma
Li et al. 2015	*Oryzaephilus mercator*	Sylvanidae	Canada	wsp,MLST	n.n.	n.n.	n.n.	n.n.	n.n.	n.n.	n.n.	n.n.	n.n.
Sharaf et al. 2010	*Oryzaephilus surinamensis*	Sylvanidae	Israel	16S	n.n.	2	94	69-86% infected	n.n.	n.n.	n.n.	possible CI	n.n.
Sharaf et al. 2013	*Oryzaephilus surinamensis*	Sylvanidae	Israel	16S	COI,16S	3	n.n.	n.n.	n.n.	n.n.	n.n.	n.n.	n.n.
Li et al. 2015	*Oryzaephilus surinamensis*	Sylvanidae	Canada,Europe,USA	wsp,MLST	n.n.	5	n.n.	all infected	n.n.	n.n.	n.n.	n.n.	n.n.
Kageyama et al. 2010	*Oryzaephilus surinamensis*	Sylvanidae	Japane	wsp	n.n.	n.n.	n.n.	n.n.	n.n.	n.n.	n.n.	n.n.	n.n.
Kageyama et al. 2010	*Tribolium confusum*	Tenebrionidae	Japane	wsp	n.n.	n.n.	n.n.	n.n.	n.n.	n.n.	n.n.	n.n.	n.n.
Goodacre et al. 2015	*Tribolium confusum*	Tenebrionidae	USA, China, Uganda	wsp	COI	9	n.n.	n.n.	n.n.	n.n.	n.n.	n.n.	n.n.
Li et al. 2016	*Tribolium confusum*	Tenebrionidae	Canada	wsp	n.n.	colonies	80	all infected?	100% infected?	n.n.	n.n.	CI	n.n.
Ming et al. 2015	*Tribolium confusum*	Tenebrionidae	China	wsp	EF-2	1	numerous	n.n.	n.n.	n.n.	n.n.	CI	n.n.
Fialho & Stevens 1997	*Tribolium confusum*	Tenebrionidae	Asia, USA, Europe	ftsZ,ITS	n.n.	11	-	all infected	all infected	n.n.	B	n.n.	n.n.
Fialho &Stevens 1996	*Tribolium confusum*	Tenebrionidae	Asia, USA, Europe	16S	n.n.	8 strains	n.n.	n.n.	n.n.	n.n.	n.n.	CI	n.n.
O'Neill et al. 1992	*Tribolium confusum*	Tenebrionidae	n.n.	16S	n.n.	n.n.	n.n.	n.n.	n.n.	n.n.	n.n.	n.n.	n.n.
Li et al. 2015	*Tribolium confusum*	Tenebrionidae	Canada,Europe,USA	wsp,MLST	n.n.	16	n.n.	15/16 populations infected	n.n.	n.n.	n.n.	n.n.	n.n.
Fialho & Stevens 2000	*Tribolium madens*	Tenebrionidae	n.n.	ftsZ,wsp,ITS,groEL	n.n.	3 strains	n.n.	2 strains infected	n.n.	n.n.	n.n.	male-killing	n.n.

Abbreviations: W. – *Wolbachia* , LD - linkage disequilibrium, SS- selective sweep, CI - cytoplasmic incompatibility, MLST – multilocus sequence typing, gatB - aspartyl/glutamyl-tRNA(Gln) amidotransferase, subunit B, coxA - cytochrome c oxidase, subunit I, hcpA - conserved hypothetical protein, ftsZ - cell division protein, fbpA - fructose-bisphosphate aldolase, wsp – *Wolbachia* surface protein, 16S/18S/28S rDNA - 16S/18S/28S ribosomal DNA, groEL - heat-shock protein 60, COI – cytochrome oxidase subunit I, ITS – internal transcribed spacer, EF1α – elongation factor 1α, Wg - wingless, H3 - histonr H3, msats – microsatellites.

References to Supplementary table 1.

Aikawa T, Anbutsu H, Nikoh N, Kikuchi T, Shibata F, Fukatsu T. 2009. Longicorn beetle that vectors pinewood nematode carries many *Wolbachia* genes on an autosome. *Proceedings of the Royal Society B: Biological Sciences* **276**: 3791–3798. DOI 10.1098/rspb.2009.1022.

Arthofer W, Riegler M, Avtzis D, Stauffer C. 2009. Evidence for low-titre infections in insect symbiosis: Wolbachia in the bark beetle Pityogeneschalcographus (Coleoptera, Scolytinae). Environmental Microbiology **11**: 1923–33. DOI: 10.1111/j.1462-2920.2009.01914.x.

Barr KL, Hearne LB, Briesacher S, Clark TL, Davis GE. 2010. Microbial Symbionts in Insects Influence Down-Regulation of Defense Genes in Maize. *PLoS ONE* **5**: e11339. DOI: 10.1371/journal.pone.0011339.

Berasategui A, Axelsson K, Nordlander G, Schmidt A, Borg-Karlson A-K, Gershenzon J, Terenius O, Kaltenpoth M. 2016. The gut microbiota of the pine weevil is similar across Europe and resembles that of other 649 conifer beetles. *Molecular Ecology* **25:** 4014–4031. DOI: 10.1111/mec.14186.

Bili M, Cortesero AM, Mougel C, Gauthier JP, Ermel G, Simon JC, Outreman Y, Terrat S, Mahéo F, Poinsot D. 2016. Bacterial Community Diversity Harboured by Interacting Species. *PLoS ONE* **11**: e0155392. DOI:10.1371/journal.pone.0155392.

Campbell BC, Bragg TS, Turner C E. 1992. Phylogeny of symbiotic bacteria of four weevil species (Coleoptera: Curculionidae) based on analysis of 16s ribosomal DNA. *InsectBiochemistry and Molecular Biology* **22**: 415–421. DOI 10.1016/0965-1748(92)90136-3.

Carvalho GA, Correa AS, Oliveira LO, Guedes RNC. 2014. Evidence of horizontal transmission of primary and secondary endosymbionts between the maize and rice weevils (Sitophilus zeamais and S.oryzae) and the parasitoid Theocolax elegans. *Journal of Stored Products Research* **59**: 61–65. DOI: 10.1016/j.jspr.2014.05.004.

Chafee ME, Funk DJ, Harrison RG, Bordenstein SR. 2010. Lateral phage transfer in obligate intracellular bacteria (Wolbachia): Verification from natural populations. *Molecular Biology and Evolution* **27**:501—505. DOI: 10.1093/molbev/msp275.

Chen SJ, Lu F, Cheng JA, Jiang MX, Way MO. 2012. Identification and Biological Role of the Endosymbionts *Wolbachia* in Rice Water Weevil (Coleoptera: Curculionidae). *Environmental Entomology* **41**: 469–477. DOI 10.1603/EN11195.

Clark ME, Bailey-Jourdain C, Ferree PM, England SJ, Sullivan W, Windsor DM, Werren JH. 2008. Wolbachia modification of sperm does not always require residence within developing sperm. *Heredity* **101**: 420–428. DOI: 10.1038/hdy.2008.71.

Clark TL, Meinke LJ, Skoda SR, Foster JE. 2001. Occurrence of *Wolbachia* in Selected Diabroticite (Coleoptera: Chrysomelidae) Beetles. *Annals of the Entomological Society of America* **94**: 877–885.

Dudek K, Humińska K, Wojciechowicz J, Tryjanowski P. 2017. Metagenomic survey of bacteria associated with the invasive ladybird *Harmonia axyridis* (Coleoptera: Coccinellidae). *European Journal of Entomology* **114**: 312–316. DOI 10.14411/eje.2017.038.

Elnagdy S, Majerus MEN, Gardener M, Lawson-Handley L. 2013. The direct effects of male-killer infection on fitness of ladybird hosts (Coleoptera: Coccinellidae). *Journal of Evolutionary Biology* **26**:1816–1825. DOI 10.1111/jeb.12186.

Fialho RF, Stevens L. 1996. *Wolbachia* infections in the flour beetle *Tribolium confusum* : evidence for a common incompatibility type across strains. *Journal of Invertebrate Pathology* **67**: 195–197. DOI 10.1006/jipa.1996.0032,

Fialho RF, Stevens L. 1997. Molecular evidence for single *Wolbachia* infections among geographic strains of the flour beetle *Tribolium confusum* . *Proceedings of the Royal Society B: Biological Sciences* **264**: 1065–1068. DOI 10.1098/rspb.1997.0147.

Fialho RF, Stevens L. 2000. Male-killing Wolbachia in a flour beetle. *Proceedings of the Royal Society B: Biological Sciences* **267**: 1469–1474. DOI 10.1098/rspb.2000.1166.

Floate KD, Coghlin PC, Dosdall L. 2011. A Test using *Wolbachia* Bacteria to Identify Eurasian Source Populations of Cabbage Seedpod Weevil, *Ceutorhynchus obstrictus* (Marsham), in North America. *Environmental Entomology* **40**: 818–823. DOI 10.1603/EN10315.

Frank JH, Erwin TL & Hemenway RC. 2009. Economically Beneficial Ground Beetles. The specialized predators *Pheropsophus aequinoctialis* (L.) and *Stenaptinus jessoensis* (Morawitz): Their laboratory behavior and descriptions of immature stages (Coleoptera, Carabidae, Brachininae). *Zookeys* **14**: 1-36. DOI: 10.3897/zookeys.14.188.

García-Vázquez D, Ribera I. 2016. The origin of widespread species in a poor dispersing lineage (diving beetle genus Deronectes). *PeerJ* **4**: e2514. DOI: 10.7717/peerj.2514.

Giordano R, Jackson JJ, Robertson HM. 1997. The role of *Wolbachia* bacteria in reproductive incompatibilities and hybrid zones of *Diabrotica* beetles and *Gryllus* crickets. *Proceedings of the National Academy of Sciences of the USA* **94**: 11439–11444.

Goodacre SL, Fricke C, Martin OY. 2015. A screen for bacterial endosymbionts in the model organisms *Tribolium castaneum, T.confusum, Callosobruchus maculatus* , and related species. *Insect Science* **22**: 165-177. DOI: 10.1111/1744-7917.12096.

Goryachevaa II, Blekhmanb AV, Andrianova BV, Gorelovaa TV, Zakharova IA. 2015. Genotypic Diversity of *Wolbachia* pipientis in Native and Invasive *Harmonia axyridis* Pall., 1773 (Coleoptera, Coccinellidae) Populations. *Russian Journal of Genetics* **8**: 731–736.

Heddi A, Grenier AM, Khatchadourian Ch, Charles H, Nardon P. 1999. Four intracellular genomes direct weevil biology: Nuclear, mitochondrial, principal endosymbiont, and *Wolbachia* . *Proceedings of the National Academy of Sciences of the USA* **96**: 6814–6819. DOI 10.1073/pnas.96.12.6814.

Heintzman PD, Elias SA, Moore K, Paszkiewicz K, Barnes I. 2014. Characterizing DNA preservation in degraded specimens of *Amara alpina* (Carabidae: Coleoptera). *Molecular Ecology Resources* **14**: 606–615. DOI 10.1111/1755-0998.12205.

Hurst GDD, Jiggins FM, von der Schulenburg JHG, Bertrand D, West SA, Goriacheva II, Zakharov IA, Werren JH, Stouthamer R, Majerus MEN. 1999b. Male-killing *Wolbachia* in two species of insect. *Proceedings of the Royal Society B: Biological Sciences* **266**: 735–740. DOI 10.1098/rspb.1999.0698.

Iwase S, Tani S, Saeki Y, Tuda M, Haran J, Skuhrovec J, Takagi M. 2015. Dynamics of infection with Wolbachia in Hyperapostica (Coleoptera: Curculionidae) during invasion and establishment. *Biological Invasions* **17**: 3639–3648. DOI: 10.1007/s10530-015-0985-1.

Jäckel R, Mora D, Dobler S. 2013. Evidence for selective sweeps by *Wolbachia* infections: Phylogeny of *Altica* leaf beetles and their reproductive parasites. *Molecular Ecology* **22**: 4241–4255. DOI 10.1111/mec.12389.

Jeyaprakash A, Hoy MA 2000. Long PCR improves *Wolbachia* DNA amplification: wsp sequences found in 76% of 63 arthropod species. *Insect Molecular Biology* **9**: 393–405. DOI 10.1046/j.1365-2583.2000.00203.x.

Jeong G, Kang T, Park H, Choi J, Hwang S, Kim W, Choi Y, Lee K, Park I, Sim H 2009. *Wolbachia* infection in the Korean endemic firefly, *Luciola unmunsana* (Coleoptera: Lampyridae). *Journal of Asia-Pacific Entomology* **12**:33–36. DOI 10.1016/j.aspen.2008.11.001.

Kageyama D, Narita S, Imamura T, Miyanoshita A. 2010. Detection and identification of Wolbachiaendosymbionts from laboratory stocks of stored-product insect pests and their parasitoids. *Journal of Stored Products Research* **46**: 13–19. DOI: 10.1016/j.jspr.2009.07.003.

Kajtoch Ł, Korotyaev B, Lachowska-Cierlik D. 2012. Genetic distinctness of parthenogenetic forms of European *Polydrusus* weevils of the subgenus *Scythodrusus* . *Insect Science* **19**: 183–194. DOI 10.1111/j.1744-7917.2011.01448.x.

Kajtoch Ł, Montagna M, Wanat M. 2017. Species delimitation within the Bothryorrhynchapion weevils: multiple evidence from genetics, morphology and ecological associations. *Molecular Phylogenetics and Evolution* . DOI: 10.1016/j.ympev.2017.12.022.

Kawasaki Y, Schuler H, Stauffer C, Lakatos F, Kajimura H. 2016. *Wolbachia* endosymbionts in haplodiploid and diploid scolytine beetles (Coleoptera: Curculionidae: Scolytinae). *Environmental Microbiology Reports* **8**: 680–688. DOI 10.1111/1758-2229.12425.

Keller GP, Windsor DM, Scucedo JM, Werren JH. 2004. Reproductive effects and geographical distributions of two *Wolbachia* strains infecting the Neotropical beetle, *Chelymorpha alternans* Boh. (Chrysomelidae, Cassidinae). *Molecular Ecology* **13**: 2405–2420. DOI 10.1111/j.1365-294X.2004.02213.x.

Kittayapong P, Jamnongluk W, Thipaksorn A, Milne JR, Sindhusake C. 2003. *Wolbachia* infection complexity among insects in the tropical rice-field community. *Molecular Ecology* **12**: 1049–1060. DOI:10.1046/j.1365-294X.2003.01793.x

Kolasa M, Montagna M, Mereghetti V, Kubisz D, Mazur MA, Kajtoch Ł. 2017. Preliminary evidence of *Wolbachia* horizontal transmission between *Crioceris* leaf beetles and *Asparagus* host plants. *European Journal of Entomology* **114**: 446–454. DOI 10.14411/eje.2017.057.

Kondo N, Ijichi N, Shimada M, Fukatsu T. 2002. Prevailing triple infection with *Wolbachia* in *Callosobruchus chinensis* (Coleoptera: Bruchidae). *Molecular Ecology* **11**: 167–180. DOI 10.1046/j.0962-1083.2001.01432.x.

Kondo N, Shimada M, Fukatsu T. 1999. High prevalence of *Wolbachia* in the azuki bean beetle *Callosobruchus chinensis* (Coleoptera, Bruchidae). *Zoological Science* **16**: 955–962. DOI 10.2108/zsj.16.955.

Kondo N, Shimada M, Fukatsu T. 2005. Infection density of *Wolbachia* endosymbiont affected by co-infection and host genotype. *Biology Letters* **1**: 488–491. DOI 10.1098/rsbl.2005.0340.

Kondo N, Tuda M, Toquenaga Y, Lan YC, Buranapanichpan S, Horng SB, Shimada M, Fukatsu T. 2011.*Wolbachia* infections in world populations of bean beetles (Coleoptera: Chrysomelidae: Bruchinae) infesting cultivated and wild legumes. *Zoological Science* **28**: 501–508. DOI 10.2108/zsj.28.501

Kubisz D, Kajtoch Ł, Mazur MA, Lis A, Holecová M. 2012. Conservation genetics of highly isolated populations of xerothermic *Crioceris quatuordecimpunctata* (Coleoptera: Chrysomelidae). *Invertebrate Biology* **131**: 333–344. DOI 10.1111/j.1744-7410.2012.00276.x.

Lachowska-Cierlik D, Kajtoch Ł, Knutelski S. 2010. Occurrence of *Wolbachia* in central European weevils: correlations with host systematics, ecology and biology. *Entomologia Experimentalis et Applicata* **14**: 105–118. DOI 10.1111/j.1570-7458.2010.00974.x.

Li YY, Fields PG, Pang B, Coghlin PC, Floate KD. 2015. Prevalence and diversity of Wolbachia bacteria infecting insect pests of stored products. *Journal of Stored Products Research* **62**: 93-100. DOI: 10.1016/j.jspr.2009.07.003.

Li YY, Fields PG, Pang BP, Floate KD. 2016. Effects of tetracycline and rifampicin treatmens on the fecundity of the *Wolbachia* -infected host, *Tribolium confusum* (Coleoptera: Tenebrionidae). *Journal of Economic Entomology* **109**: 1458–1464. DOI 10.1093/jee/tow067.

Lu F, Kang XY, Lorenz G, Espino L, Jiang MX, Way MO 2014. Culture-independent analysis of bacterial communities in the gut of rice water weevil (Coleoptera: Curculionidae). *Annals of the Entomological Society of America* **10**: 592–600. DOI 10.1603/AN13145.

Majerus MEN, Majerus TMO. 2000. Multiple causes of male-killing in a single sample of the 2-spot ladybird, *Adalia bipunctata* (Coleoptera:Coccinellidae) from Moscow. *Heredity* **84**, 605–609. DOI 10.1046/j.1365-2540.2000.00710.x

Malloch G, Fenton B, Butcher RD. 2000. Molecular evidence for multiple infections of a new subgroup of *Wolbachia* in the European raspberry beetle *Byturus tomentosus* . *Molecular Ecology* **9**: 77–90.

Mariño Y, Verle Rodrigues JC, Bayman P. 2017.*Wolbachia* Affects Reproduction and Population Dynamics of the Coffee Berry Borer (*Hypothenemus hampei*): Implications for *Biological Control Insects* **8**: 8. DOI 10.3390/insects8010008.

Mazur M.A., Holecová M., Lachowska-Cierlik D., Lis A., Kubisz D., Kajtoch Ł. 2016. Selective sweep of *Wolbachia* and parthenogenetic host genomes on the example of the weevil *Eusomus ovulum* . *Insect Molecular Biology* **25**: 701–711. DOI 10.1111/imb.1225.

Mazur MA, Kubisz D, Kajtoch Ł. 2014. Restricted geographic distribution and low genetic distinctiveness of steppic *Crioceris quinquepunctata* (Coleoptera: Chrysomelidae) populations in central-east Europe. *Entomologica Fennica* **25**: 103–111.

Ming QL, Shen JF, Cheng C., Liu CM, Feng ZJ. 2015.*Wolbachia* infection dynamics in *Tribolium confusum* (Coleoptera: Tenebrionidae) and their effects on host mating behavior and reproduction. *Journal of Economic Entomology* **108**: 1408–1415. DOI 10.1093/jee/tov053.

Montagna M, Chouaia B, Sacchi L, Porretta D, Martin E, Giorgi A, Lozzia GC, Epis SA.2014. New Strain of *Wolbachia* in an Alpine Population of the Viviparous *Oreina cacaliae* (Coleoptera: Chrysomelidae). *Environmental Entomology* **43**: 913–922. DOI 10.1603/EN13228.

Nikoh N, Tanaka K, Shibata F, Kondo N, Hizume M, Shimada M, Fukatsu T. 2008.*Wolbachia* genome integrated in an insect chromosome: evolution and fate of laterally transferred endosymbiont genes. *Genome Research* **18**: 272–280. DOI 10.1101/gr.7144908.

Nirgianaki A, Banks GK, Frohlich DR, Veneti Z, Braig HR, Miller TA, Bedford ID, Markham PG, Savakis C, Bourtzis K 2003. Wolbachia Infections of the Whitefly Bemisia tabaci. *Current Microbiology* **47**: 0093–0101. DOI:10.1007/s00284-002-3969-1

Numajiri Y, Kondo NI, Toquenaga Y. 2017. Melanic mutation causes a fitness decline in bean beetles infected by Wolbachia. *Entomologia Experimentalis et Applicata* **164**: 54-65. DOI: 10.1111/eea.12588.

O'Neill SL, Giordano R, Colbert AM, Karr TL, Robertson HM. 1992. 16S rRNA phylogenetic analysis of the bacterial endosymbionts associated with cytoplasmic incompatibity in insects. *Proceedings of National Academy of Sciences of the USA* **89**: 2699–2702.

Okayama K, Katsuki M, Sumida Y, Okada K. 2016. Costs and benefits of symbiosis between a bean beetle and *Wolbachia* . *Animal Behaviour* **119**: 19–26. DOI 10.1016/j.anbehav.2016.07.004.

Pankewitz F, Zöllmer A, Hilker M, Gräser Y. 2007. Presence of Wolbachia in Insect Eggs Containing Antimicrobially Active Anthraquinones. *Microbial Ecology* **54**: 713–721. DOI: 10.1007/s00248-007-9230-5.

Perotti MA, Young DK, Braig HR. 2016. The ghost sex-life of the paedogenetic beetle *Micromalthus debilis* . *Scientific reports* **6:** 27364. DOI: 10.1038/srep27364.

Piper RW, Compton SG, Rasplus JY, Piry S. 2001. The species status of *Cathormiocerus britannicus* , an endemic, endangered British weevil. *Biological Conservation* **101**: 9–13. DOI 10.1016/S0006-3207(01)00048-9.

Pourali P, Roayaei Ardakani M, Jolodar A, Razi Jalali MH. 2009. PCR screening of the *Wolbachia* in some arthropods and nematodes in Khuzestan province. *Iranian Journal Of Veterinary Research* **10**:216-222.

Prakash BM & Puttaraju HP. 2006. Wolbachia endosymbiont in some insect pests of sericulture. *Current Science* **90:** 1671-1674.

Rodriguero MS, Confalonieri VA, Guedes JVC, Lanteri AA. 2010a.*Wolbachia* infection in the tribe Naupactini (Coleoptera, Curculionidae): association between thelytokous parthenogenesis and infection status. *Insect Molecular Biology* **19**: 631–640. DOI 10.1111/j.1365-2583.2010.01018.x.

Rodriguero MS, Lanteri AA, Confalonieri VA. 2010b. Mito-nuclear genetic comparison in a *Wolbachia* infected weevil: insights on reproductive mode, infection age and evolutionary forces shaping genetic variation. *BMC Evolutionary Biology* **10**:340. DOI 10.1186/1471-2148-10-340.

Roehrdanz R & Levine E 2007. *Wolbachia* bacterial infections linked to mitochondrial DNA reproductive isolation among populations of northern corn rootworm (Coleoptera: Chrysomelidae). *Annals of the Entomological Society of America* **100**: 522–531. DOI 10.1603/0013-8746(2007)100[522:WBILTM]2.0.CO;2.

Roehrdanz R, Olson D, Bourchier R, Sears S, Cortilet A, Fauske G 2006. Mitochondrial DNA diversity and *Wolbachia* infection in the flea beetle *Aphthona nigriscutis* (Coleoptera: Chrysomelidae): an introduced biocontrol agent for leafy spurge. *Biological Control* **37**:1–8. DOI 10.1016/j.biocontrol.2005.12.004.

Roehrdanz RL, Wichmann SGS. 2013. *Wolbachia* wsp gene clones detect the distribution of *Wolbachia* variants and wsp hypervariable regions among 160 individuals of a multistrain infected population of *Diabrotica barberi* (Coleoptera: Chrysomelidae). *Annals of the Entomological Society of America* **106**:329–338. DOI 10.1603/AN12118

Sharaf K, Hadid Y, Pavliček T, Nevo E. 2013. Local genetic population divergence in a saw-toothed grain beetle, *Oryzaephilus surinamensis* (L.) (Coleoptera, Cucujidae). *Journal of Stored Products Research* **53**:72–76.

Sharaf K, Horovă L, Pavliček T, Nevo E, Bureš P. 2010. Genome size and base composition in *Oryzaephilius surinamensis* (Coleoptera: Sylvanidae) (Cikeiotera: Sylvanidae) and differences between native (feral) and silo pest populations in Israel. *Journal of Stored Products Research* **46:** 34–37.

Sintupachee S, Milne JR, Poonchaisri S, Baimai V, Kittayapong P. 2006. Closely Related *Wolbachia* Strains within the Pumpkin Arthropod Community and the Potential for Horizontal Transmission via the Plant. *Microbial Ecology* **51**: 294–301. DOI:10.1007/s00248-006-9036-x

Son Y, Luckhart S, Zhang X, Lieber MJ, Lewis EE. 2008. Effects and implications of antibiotic treatment on *Wolbachia* - infected vine weevil (Coleoptera: Curculionidae). *Agricultural and Forest Entomology* **10**: 147–155. DOI 10.1111/j.1461-9563.2008.00369.x.

Sontowski R, Bernhard D, Bleidorn C, Schlegel M, Gerth M. 2015.*Wolbachia* distribution in selected beetle taxa characterized by PCR screens and MLST data. *Ecology and Evolution* **5**:4345–4353. DOI 10.1002/ece3.1641.

Stenberg, P., Lundmark, M., 2004. Distribution, mechanisms and evolutionary significance of clonality and polyploidy in weevils. *Agricultural and Forest Entomology* **6**: 259–266. DOI 10.1111/j.1461-9555.2004.00231.x.

Takano SI, Tuda M, Takasu K, Furuya N, Imamura Y, Kim S, Tashiro K, Iiyama K, Tavares M, Amaral AC. 2017. Unique clade of alphaproteobacterialendosymbionts induces complete cytoplasmic incompatibility in the coconut beetle. Proceedins of National Academy of Sciences USA **114**: 6110–6115. DOI: 10.1073/pnas.1618094114.

Toju H & Fukatsu T. 2011. Diversity and infection prevalence of endosymbionts in natural populations of the chestnut weevil: Relevance of local climate and host plants. *Molecular Ecology* **20**: 853–868. DOI: 10.1111/j.1365-294X.2010.04980.x.

Toju H, Tanabe AS, Notsu Y, Sota T, Fukatsu T. 2013. Diversification of endosymbiosis: replacements, co-speciation and promiscuity of bacteriocyte symbionts in weevils. *The ISME Journal* **7**: 1378-1390. DOI: 10.1038/ismej.2013.27.

Toševski I, Caldara R, Jović J, Hernández-Vera G, Baviera C, Gassman A, Emerson BC. 2015. Host associated genetic divergence and taxonomy in the *Rhinusa pilosa* Gyllenhal species complex: an integrative approach. *Systematic Entomology* **40**: 268–287. DOI: 10.1111/syen.12109.

Vega FE, Benavides P, Stuart J, O'Neill SL. 2002.*Wolbachia* infection in the coffee berry borer (Coleoptera: Scolytidae). *Annals of the Entomological Society of America* **95**: 374–378. DOI 10.1603/0013-8746(2002)095[0374:WIITCB]2.0.CO;2.

Weinert LA, Tinsley MC, Temperley M, Jiggins FM. 2007. Are we underestimating the diversity and incidence of insect bacterial symbionts? A case study in ladybird beetles. *Biology Letters* **3**: 678–681. DOI: 10.1098/rsbl.2007.0373.

Werren JH, Windsor D, Guo L 1995. Distribution of *Wolbachia* among neotropical arthropods. *Proceedings of the Royal Society B: Biological Sciences* **262**: 197–204. DOI 10.1098/rspb.1995.0196.

Werren JH, Windsor DM. 2000. *Wolbachia* infection frequencies in insects: evidence of a global equilibrium? *Proceedings of the Royal Society B: Biological Sciences* **1450**: 1277–1285. DOI 10.1098/rspb.2000.1139.

White JA, Richards NK, Laugraud A, Saeed A, Curry MM, McNeill MR. 2015. Endosymbiotic candidates for parasitoid defense in exotic and native New Zealand weevils. *Microbial Ecology* **70**: 274–286. DOI: 10.1007/s00248-014-0561-8.

Xue H-J, Li W-Z, Nie R-E, Yang X-K. 2011. Recent Speciation in Three Closely Related Sympatric Specialists: Inferences Using Multi-Locus Sequence, Post-Mating Isolation and Endosymbiont Data. *PLoS ONE* **6**: e27834. DOI:10.1371/journal.pone.0027834.

Yun Y, Peng Y, Liu FX, Lei C. 2011.*Wolbachia* screening in spiders and assessment of horizontal transmission between predator and prey. *Neotropical Entomology* **40**: 164–169. DOI 10.1590/S1519-566X2011000200002.

Zchori-Fein E, Borad C, Harari AR. 2006. Oogenesis in the date stone beetle, Coccotrypesdactyliperda, depends on symbiotic bacteria. *Physiological Entomology* **31:** 164–169. DOI: 10.1111/j.1365-3032.2006.00504.x.

Zhang X, Luckhart S, Tu J, Pfeiffer DG. 2010. Analysis of *Wolbachia* strains associated with *Conotrachelus nenuphar* (Coleoptera: Curculionidae) in the eastern United States. *Environmental Entomology* **39**: 396-405. DOI 10.1603/EN09276.
