# Supplementary figures and images for: Current state of knowledge on Wolbachia infection among Coleoptera: a systematic review
Source: PeerJ. 2018 Mar 9;6:e4471. doi: 10.7717/peerj.4471 (PMC5846457; doi:10.7717/peerj.4471)

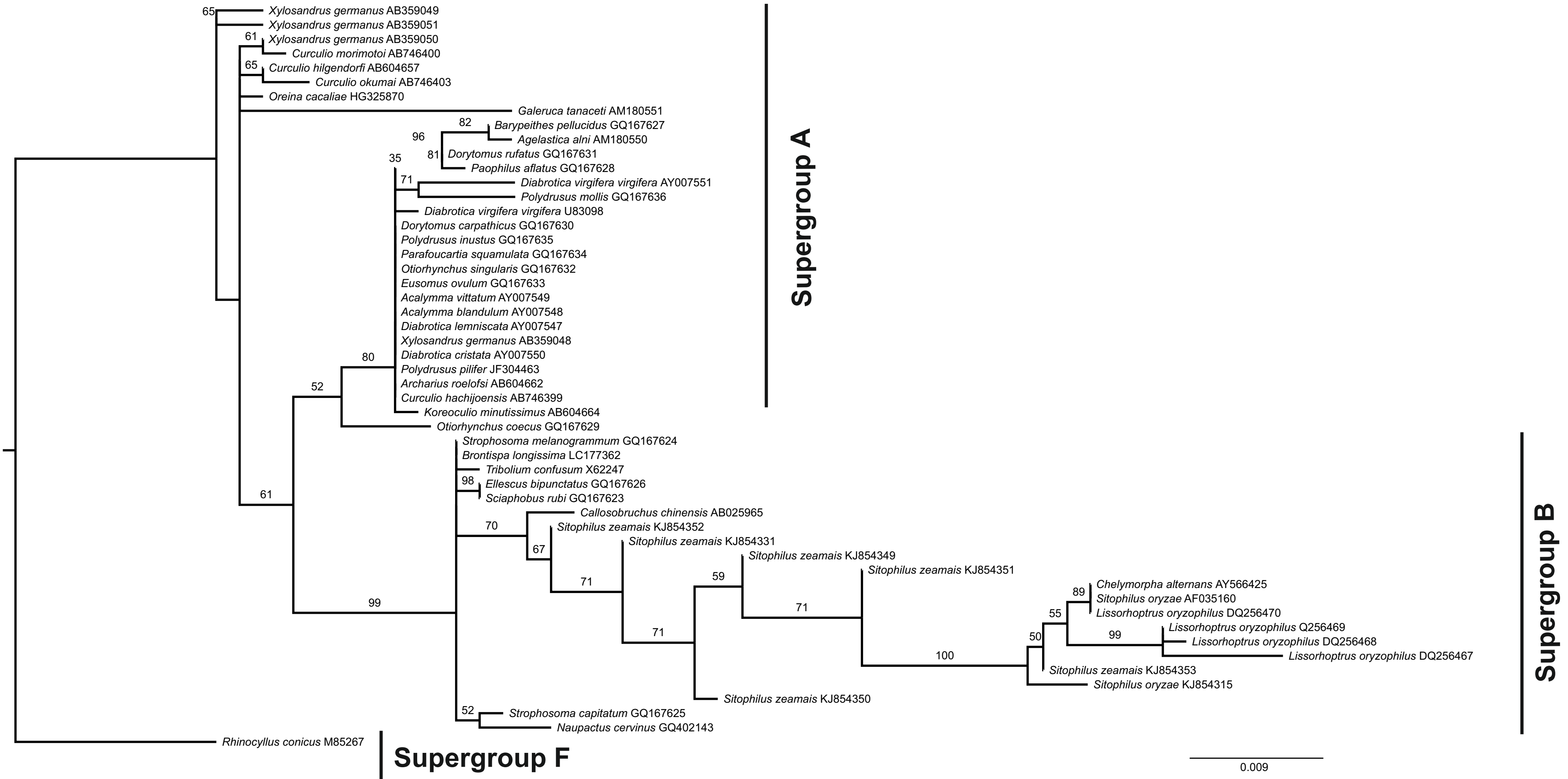

Supplement: Figure S1 — Maximum likelihood phylogenetic tree reconstructed based on Wolbachia 16S rDNA gene sequences obtained from hosts belonging to various species of Coleoptera (data downloaded from NCBI GenBank https://www.ncbi.nlm.nih.gov/genbank/). Statistical supports (bootstrap values) are presented above the branches (shown only if >0.50). [file peerj-06-4471-s002.pdf]

*Agrilus araxenus* KT199202  
*Sphaerobothris aghababiani* KT199203

Supergroup F

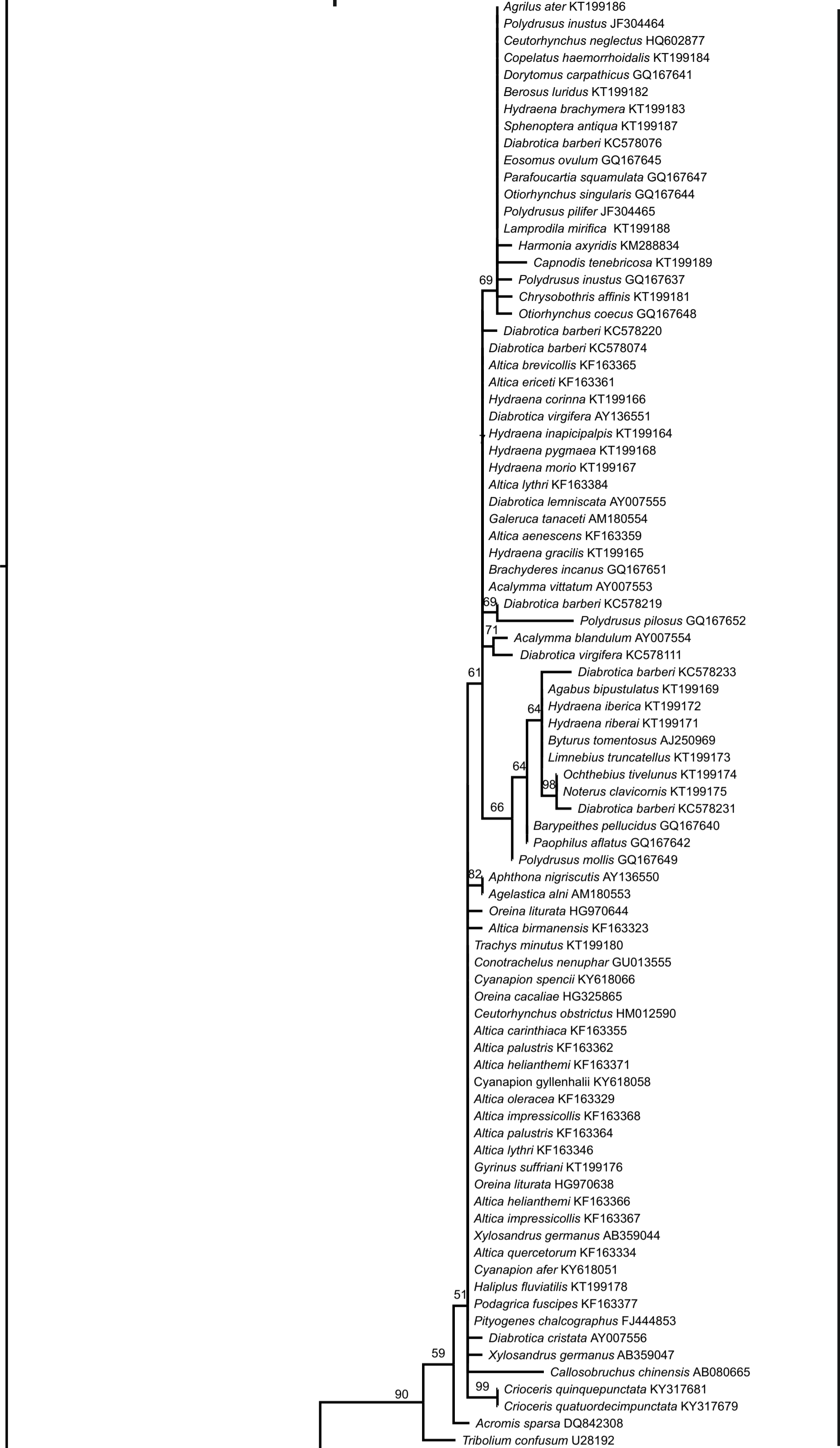

Supergroup A

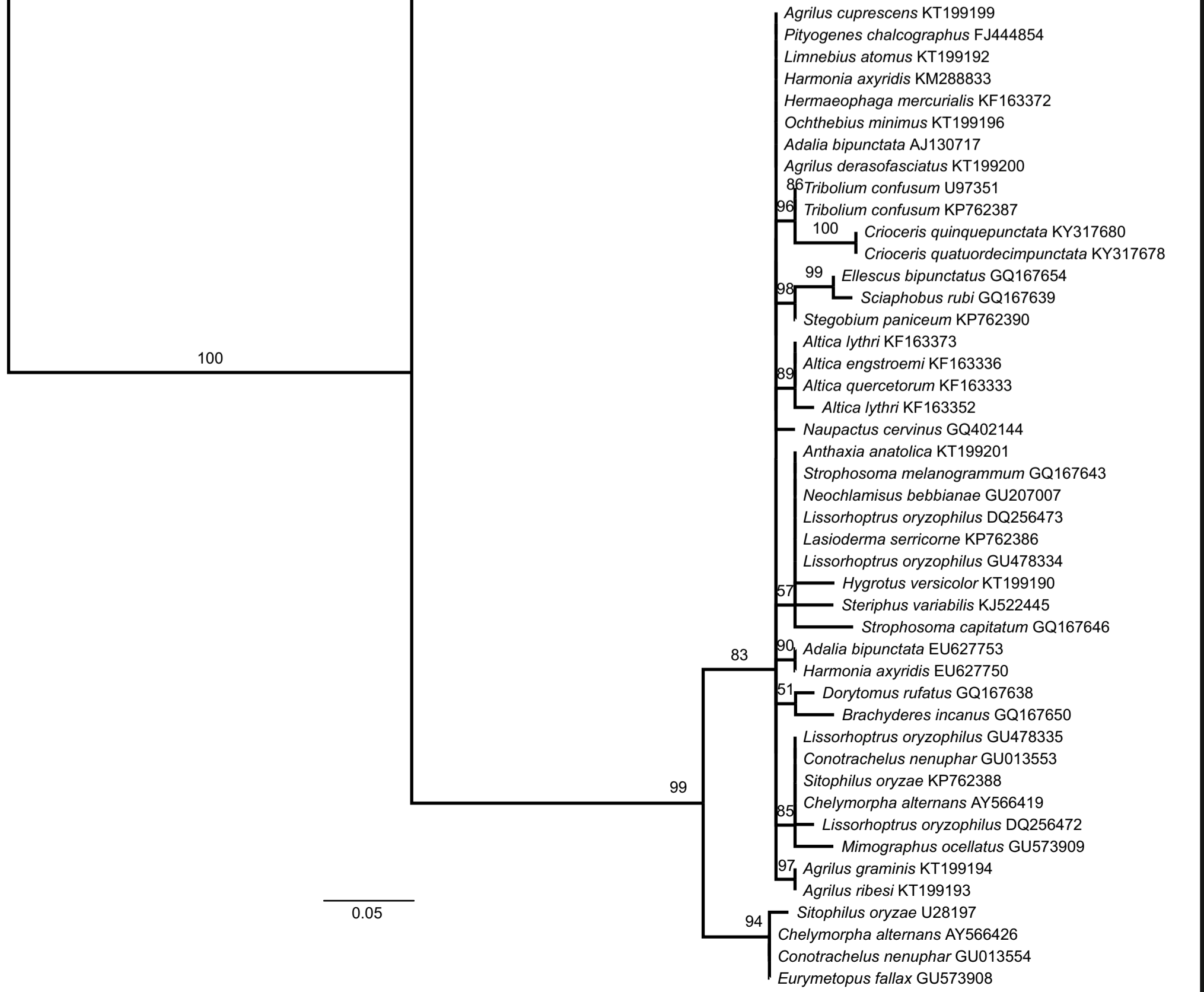

Supergroup B

Supplement: Figure S2 — Maximum likelihood phylogenetic tree reconstructed based on Wolbachia ftsZ gene sequences obtained from hosts belonging to various species of Coleoptera (data downloaded from NCBI GenBank https://www.ncbi.nlm.nih.gov/genbank/). Statistical supports (bootstrap values) are presented above the branches (shown only if >0.50). [file peerj-06-4471-s003.pdf]
